# Thermal–Mechanical and Microstructural Simulation of Rotary Friction Welding Processes by Using Finite Element Method

**DOI:** 10.3390/ma17040815

**Published:** 2024-02-08

**Authors:** Hossein Mani, Aboozar Taherizadeh, Behzad Sadeghian, Behzad Sadeghi, Pasquale Cavaliere

**Affiliations:** 1 Department of Materials Engineering, Isfahan University of Technology, Isfahan 8415683111, Iran; hossein200065@gmail.com (H.M.); a.taheri@iut.ac.ir (A.T.); behzadsadeghian91@gmail.com (B.S.); 2 Department of Innovation Engineering, University of Salento, Via per Arnesano, 73100 Lecce, Italy; behzad.sadeghi@unisalento.it

**Keywords:** rotary friction welding, finite element method, Inconel 718, microstructural simulation, Johnson–Avrami model

## Abstract

Rotary friction welding is one of the most crucial techniques for joining different parts in advanced industries. Experimentally measuring the history of thermomechanical and microstructural parameters of this process can be a significant challenge and incurs high costs. To address these challenges, the finite element method was used to simulate thermomechanical and microstructural aspects of the welding of identical superalloy Inconel 718 tubes. Numerical simulation results were used to compute essential mechanical and metallurgical parameters such as temperature, strain, strain rate, volume fraction of dynamic recrystallization, and grain size distribution. These parameters were subsequently verified using experimental test results. The Johnson–Avrami model was utilized in the microstructural simulation to convert thermomechanical parameters into metallurgical factors, employing a FORTRAN subroutine. The calculated thickness of the recrystallization zone in the wall was 480 and 850 μm at the tube wall’s center and edge, respectively. These values were reported from experimental measurements as 500 and 800 μm, respectively. The predicted grain size changes from the center to the edge of the wall thickness, near the weld interface, ranged from 2.07 to 2.15 μm, comparable to the experimental measurements ranging from 1.9 to 2.2 μm. Various curves are also presented to explore the correlation between thermomechanical and microstructural parameters, with the experimental results revealing predictable microstructure evolutions correlated with thermomechanical changes.

## 1. Introduction

**Figure 1:**
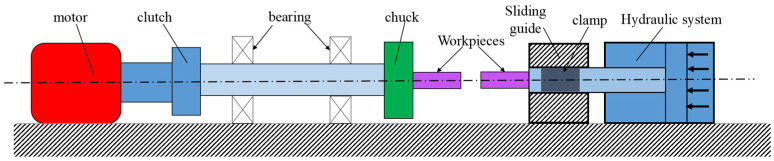
Schematic of the rotary friction welding machine.

Conventional fusion welding techniques struggle to maintain high mechanical properties due to various issues [1,2]. These issues include strain age cracking in the heat-affected zone (HAZ), micro-fissuring, formation of brittle phases, and segregation of elements like niobium and boron [3,4]. Rotary friction welding (RFW), a solid-state welding procedure, has been developed as a process to address these challenges [5,6]. RFW generates heat through friction between two surfaces undergoing severe rotation at the weld interface, coupled with the application of an axial force. This process causes the materials to plasticize and mechanically join, yielding high-quality welds with enhanced mechanical properties [7,8]. Figure 1 provides a schematic of the rotary friction welding process. This machine comprises several key components, including an electric motor, a clutch, a bearing, and a system for securing work pieces. The equipment on the left side is responsible for generating a variable and adjustable rotational speed for the fixed sample. On the right side, the hydraulic unit, clamp, and movable jack work in unison to exert a consistent pressure on the other sample. This intricate setup ensures the effective operation of the rotary friction welding process [9].

With the advancement of manufacturing technologies and the increasing demand for cost-efficiency, numerical methods are increasingly being utilized by researchers to study the RFW process. Numerical methods offer an alternative to the execution of multiple practical tests for investigating the influence of process parameters on mechanical properties of final products. A better understanding of the underlying mechanisms of the welding process is facilitated by using these methods, enabling further optimization and improvement with reducing time and expenses. The efficient optimization of welding properties, leading to stronger and more cost-effective welds, is made possible through the use of these numerical methods [10,11,12]. Fu and Duan [13] spearheaded the research in this field, focusing on coupled thermomechanical simulation for the friction welding process. Their study used simulation results, including temperature, stress, strain, and the final shape of the deformation zone, to optimize the initial parameters of the welding process [13]. Recently, Yang et al. [14] utilized the Abaqus/CAE 2022 software for a coupled temperature–mechanical finite element simulation to explore the RFW process’s efficacy in joining titanium alloys [14]. The study disclosed a consistent grain size distribution at the weld interface, corroborated by experimental data, due to a uniform temperature distribution noted as a key simulation outcome. Nonetheless, Yang et al. did not extend their predictive or experimental study of grain size distribution to the entire welded tube [14].

Khosroshahi et al. [15] implemented three validation methods for the techniques employed in the numerical simulation of the RFW process, focusing on axial shortening, flash deformation, and thermocouple temperature to verify the simulation results’ accuracy. Yet, their study did not delve into microstructural evolutions, and it recorded a maximum error of 18.6% in the thermomechanical simulation [15]. Conversely, Okeke et al. [16] examined both thermomechanical and microstructural aspects of linear friction welding for Inconel 718 alloys. They utilized the Kolmogorov–Mehl–Johnson–Avrami (JMAK) model in their microstructural simulation to assess the volume fraction and grain size evolution of dynamic recrystallization (DRX) throughout the welded zones’ tube [16]. However, the effect of thermomechanical parameters on microstructural evolution was not adequately defined through simulation in these studies.

Nelson et al. [17] conducted a comprehensive research study on the RFW process of the Inconel 718 alloy, measuring grain size and microstructure evolution at different points of the welded tube using electron beam microscopy images [17]. However, Nelson’s study could be improved by including the calculation of microstructural parameters in all parts of the welded tube using thermal–mechanical simulations and the JMAK model. The JMAK model is a phenomenological model that has been used to describe the kinetics of isothermal phase transformations since the 1940s [18]. By incorporating this model into different analyses, the researchers could gain a better understanding of the microstructural changes that occur during welding processes and of how these changes affect the properties and performance of welded parts. Overall, this would provide a more comprehensive and meaningful analysis of the welding process.

In this study, to address this gap, firstly by finite element method (FEM), a thermal–mechanical simulation was conducted. This type of simulation was performed to gain valuable insights into the distribution of stress, strain, strain rate, temperature, etc., for Inconel 718 welded using the RFW process. Secondly, JMAK constitutive equations were numerically developed using a subroutine in Abaqus to reach grain size and volume fraction of the dynamic recrystallization history. Thirdly, for each simulation, proper validations were performed to determine the accuracy of initial conditions and outcomes, or results, which is vital in simulation studies. Finally, the relationship between thermomechanical and microstructural investigation is presented.

## 2. FE Modeling Methodology

**Figure 2:**
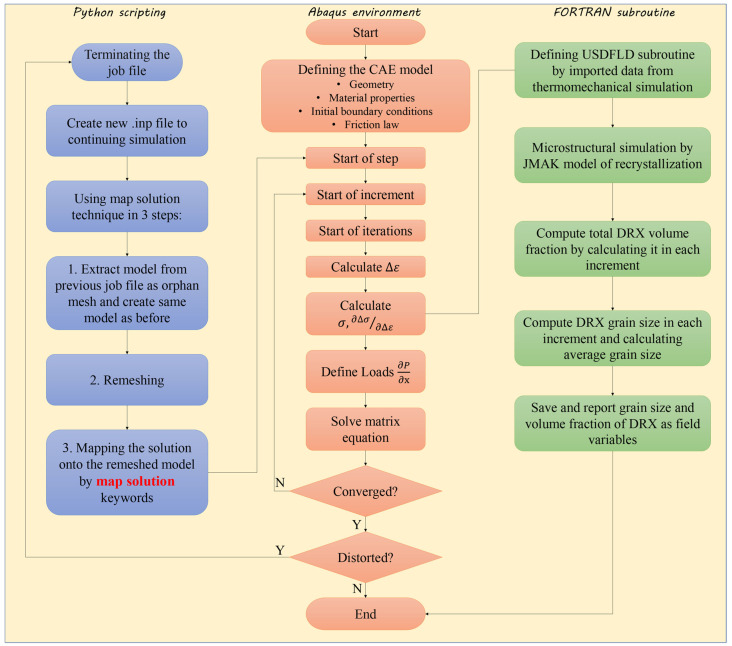
Flowchart of different parts of the multiphysics computational modeling for the RFW process used in this study.

Figure 2 delineates the multiphysics computational modeling procedure, displaying various components and the sequential coupling between thermal–mechanical (in red) and microstructural (in green) submodels. At each time step, both submodels are concurrently solved to simulate different aspects of the RFW process. Key state variables, including temperature, plastic strain, and strain rate, are determined using the Abaqus/standard solver (implicit dynamic temperature–displacement). The computed results are then integrated by the USDFLD subroutine for the microstructure model. Subsequently, the JMAK equations are resolved using Abaqus/standard, aligned with the associated USDFLD subroutine. A crucial assumption in the sequential coupling of the submodels is that the dynamic recrystallization (DRX) of γ grains remains inconsequential to the thermomechanical properties of IN718 during the RFW process [19]. The nature of numerical algorithms and formulations in the coupled FE simulations, similar to the ones used in the current study, makes it possible to calculate the cross effects of different parameters in the course of time steps of the problem solving. Also, a remeshing technique was employed to counter the severe distortion of elements due to significant plastic deformation in this simulation, facilitated by Python scripting. The complete methodology, executed in an Abaqus environment utilizing Python scripting and a FORTRAN subroutine, is succinctly represented in the flowchart below.

### 2.1. Thermal–Mechanical Constitutive Equations

In the rotary friction welding process, the frictional heat generated at the weld interface between two pieces accounts for approximately 85–90% of the overall generated heat [20]. This heat can be computed using Amonton’s law, as shown in Equation (1) [20,21]. The remaining 10–15% of the total heat generated in this process is attributed to the heat generated from plastic deformation, which can be quantified by Equation (2) [20,22,23]. These Computations yield valuable insights throughout the welding, enabling refined prognostications of material properties into the materials’ thermal behavior and efficacy.

(1) $$Q_{f}=\mu P\omega r$$

(2) $$Q_{p}=\sigma {\dot{\varepsilon }}^{pl}$$

In the equations mentioned above, $Q_{f}$ and $Q_{P}$ represent the heat generated by friction and plastic deformation, respectively. These values can be calculated using various parameters such as the equivalent plastic strain rate (${\dot{\varepsilon }}^{pL}$), axial pressure (*P*), rotational speed (*ω*), friction coefficient (*µ*), specimen radius (*r*), and stress (*σ*) [22,24]. In this simulation, the inelastic heat fraction, denoted by *η*, is postulated to be 0.9. By understanding the distribution of heat generated during the welding process, engineers and researchers can optimize welding parameters and improve the quality of welded joints.

**Figure 3:**
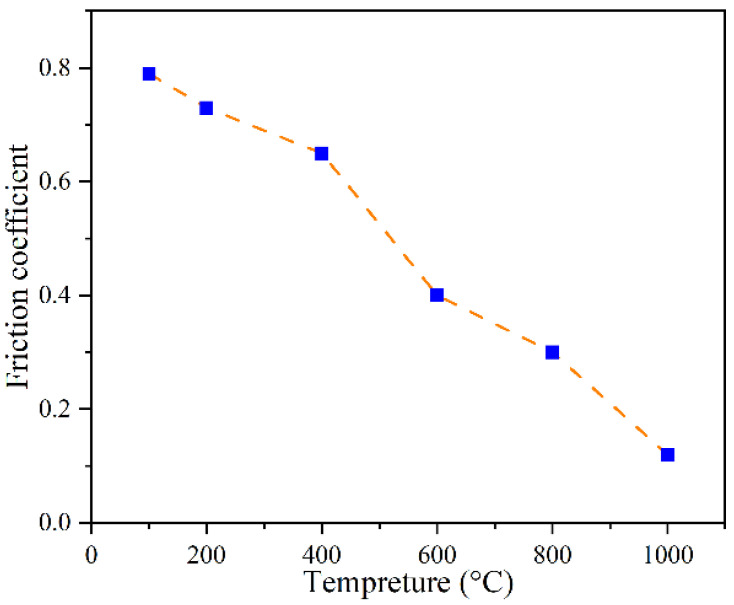
The relationship between the friction coefficient and the temperature (data from [27]).

In the simulation of heat generation during the RFW process, accurately calculating temperature changes is crucial. Based on the non-equilibrium Fourier’s law, temperature changes within the rotated friction-welded tube can be calculated by using a proper coordinate system, as depicted in Equation (3) [20,22]. This calculation provides valuable insights into the thermal behavior of materials during the welding process and allows for more accurate predictions of material properties and performance.

(3) $$\rho c\frac{\partial T}{\partial t}=Q+k^{\prime }\left[\frac{\partial^{2}T}{\partial^{2}x}+\frac{\partial^{2}T}{\partial^{2}y}+\frac{\partial^{2}T}{\partial^{2}z}\right]$$

where the coefficient of thermal conductivity is *k*′, the density is ρ, the heat capacity is c, *t* is time, and *T* is temperature [20]. To calculate the heat generation in weld interfaces, establishing friction conditions in contact interfaces is essential. Under sliding conditions, Coulomb’s law, expressed as Equation (4), was used for numerical computations of the friction coefficient [15,25,26].

(4) $$\tau_{f}=\mu P$$

where the friction coefficient is μ, the contact pressure is *P*, and contact shear stress is $\tau_{f}$. Bai et al. [27] conducted experiments to measure the friction coefficient of Inconel 718 at different temperatures. Their results, depicted in Figure 3, were used in the current simulation for accurate analysis.

### 2.2. Microstructural Constitutive Equations

To study the microstructural evolutions associated with dynamic recrystallization and nucleation during hot deformation processes, Avrami’s kinetic equations have been extensively utilized [16,28,29,30,31,32]. The onset of dynamic recrystallization is identified when the accumulated plastic strain exceeds the critical strain threshold and the development of recrystallization can be assessed using strain changes at each time step within the elements. Predominately, research studies have utilized Equation (5) to represent the volume fraction of dynamic recrystallization, offering valuable insights into the behavior of materials during hot deformation processes [29,32].

(5) $$X_{DRX}=1-\exp\left(-k_{D}{\left(\frac{\varepsilon -\varepsilon_{c}}{\varepsilon_{0.5}}\right)}^{n_{D}}\right)\;\;\left(\varepsilon \geq \varepsilon_{c}\right)$$

In this equation, the volume fraction of dynamic recrystallization is denoted by $X_{DRX}$, ranging between 0 and 1. The material constant values of $n_{D}$ and $K_{D}$ in this equation are −0.8676 and 1.9, respectively [29,32]. The value of *ε* can be directly derived from the thermomechanical simulation algorithm. Other studies have outlined the procedure and equations for calculating $\varepsilon_{c}$ and $\varepsilon_{0.5}$, which represent the critical strain and the strain for the 50% volume fraction of DRX, respectively [33,34,35]. The determination of $\varepsilon_{c}$ and $\varepsilon_{0.5}$ is dependent on the Zener–Hollomon parameter and is reported in the form of Equations (6)–(8) [29,32]. Equation (9) has been commonly used to evaluate the Zener–Hollomon parameter in the measurement of dynamic recrystallization kinetics in hot deformation processes [33,34,36,37].

(6) $$\varepsilon_{p}=0.0032\times {\left(Z\right)}^{0.11376}$$

(7) $$\varepsilon_{c}=0.8\times \varepsilon_{p}$$

(8) $$\varepsilon_{0.5}=0.1343\times {\left(Z\right)}^{0.0515}$$

(9) $$Z=\dot{\varepsilon }\times \exp\left(\frac{Q_{def}}{RT}\right)\;\;\;Q_{def}=437000\;\left(j/mol.K\right)$$

(10) $$D_{DRX}=1.2736723\times 10^{7}\times {\left(Z\right)}^{-0.4215}$$

In the above equation, the peak strain is represented by $\varepsilon_{p}$, Z is the Zener–Hollomon coefficient, Q_def_ is expressed as the activation energy for plastic deformation, and $\dot{\varepsilon }$ is the strain rate of the process. According to the kinetics of dynamic recrystallization, the fully recrystallized grain size (dDRX) does not rely on the initial grain size before material deformation. Rather, it is determined solely by the temperature and strain rate of the hot deformation process [36]. For the Inconel 718 alloy, the grain size resulting from dynamic recrystallization can be calculated by measuring the Zener–Hollomon parameter using Equation (10) [38]. Calculating the final grain size in all deformation zones is pivotal for understanding the performance and optimizing the RFW process [17]. Therefore, this study conducts microstructural simulations in tandem with thermomechanical simulations.

By utilizing Equation (11) the average grain size can be accurately computed at each time step, comprising both recrystallized and non-recrystallized grains [16]. Within this equation, the recrystallized grains are specified by the first set of parentheses, while the non-recrystallized grains are determined by the initial grain size which is represented by the second set of parentheses [16,17]. According to an experimental test (EBSD maps) of the RFW process [17], the initial grain size (D_°_) was found to be 4.5 μm. By conducting a numerical simulation, the impact of the initial parameters of the RFW process on the final grain size can be determined [29,38].

(11) $$D_{avg}=\left(D_{DRX}\times X_{DRX}\right)+\left(D_{{}^{\circ}}\times \left(1-X_{DRX}\right)\right)$$

### 2.3. Material Properties and Boundary Conditions

**Figure 4:**
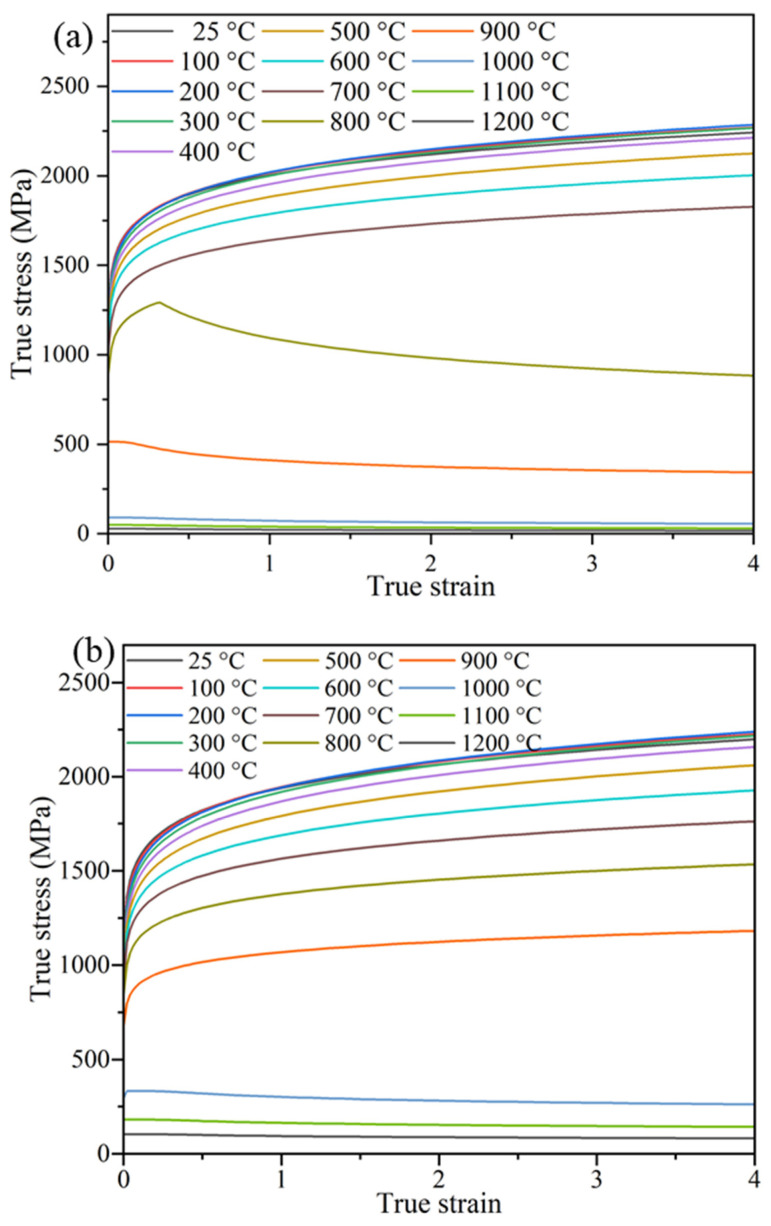
Flow stress curves of Inconel 718 alloy at strain rates of (**a**) 0.001 and (**b**) 1 (/s^−1^) for temperatures ranging from 25 to 1200 °C.

In the RFW process, the presence of severe plastic deformation under high temperatures and strain rates necessitates the accurate determination of the plastic properties of materials under various conditions (including different temperatures, strains, and strain rates). In this simulation, the JMatPro7 software [39] was utilized to obtain comprehensive information about the material properties. The accuracy of the results obtained for the Inconel 718 alloy by using the JMatPro software has been corroborated through comparisons with experimental test data [40,41,42,43]. The reliability of the material property information was ensured by a validation performed using the software. In the Abaqus software, the flow stress variations in the Inconel 718 alloy were determined over a range of temperatures, from ambient to 1200 °C, and strain rates, from 10^−5^ to 10 s^−1^. Figure 4 illustrates the changes in flow stress under strain rates of 0.001 and 1 at different temperatures.

**Figure 5:**
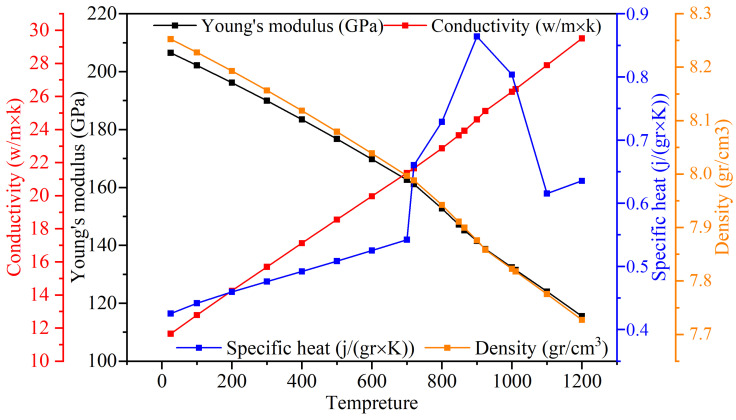
Variation in density, heat transfer coefficient, specific heat capacity, and Young’s modulus of Inconel 718 alloy vs. temperature.

To accurately simulate the RFW process, the JMatPro software was used to simulate other temperature-dependent variables such as conductivity, Young’s modulus, density, and specific heat capacity, as illustrated in Figure 5.

**Figure 6:**
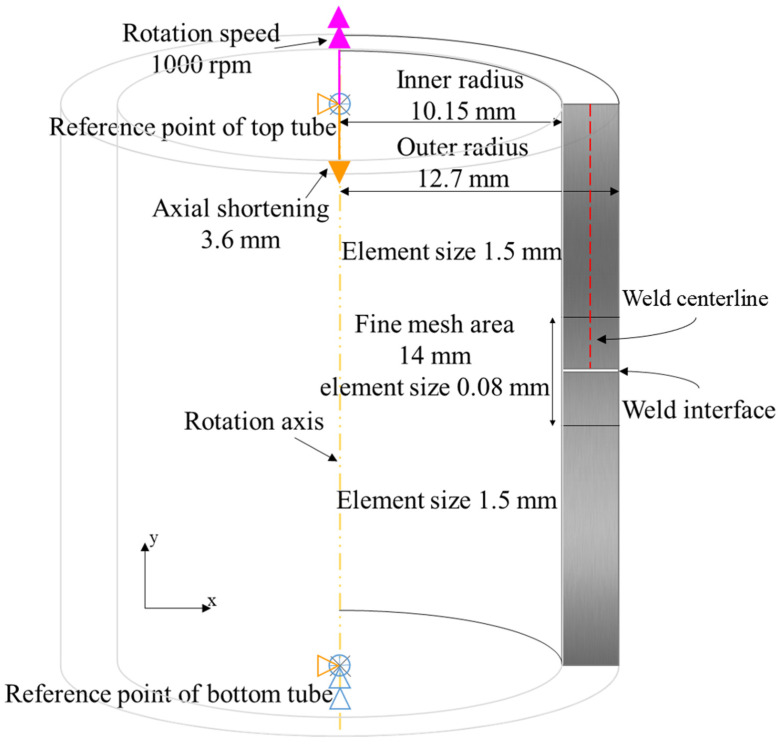
Initial dimensions and boundary conditions of the tubes in the FE model.

In this study, to ensure the accuracy of the simulation, the geometry and dimensions of the tubes were specified based on Nelson et al.’s experimental research [17]. To ensure the robustness of the simulations, different geometries including pipe-to-rod and pipe-to-pipe pieces with various thicknesses and diameters were successfully simulated, and no convergence issues or numerical errors were observed. Axisymmetric elements were used to simulate the RFW process of Inconel 718 tubes due to the symmetry of the geometry and loading of the process. As shown in Figure 6, the inner and outer radii of the tubes were 10.15 mm and 24.7 mm, respectively.

As demonstrated in Figure 2, Python scripting was applied to prevent severe distortions of the elements. To achieve this, the simulation procedure was divided into four stages. At the end of each stage, a new simulation was conducted. The final geometry from the previous mesh was extracted to the CAE mode to conduct a new stage of simulation. The boundary conditions were updated, the model was remeshed, and the solutions (field variables) were mapped onto the remeshed model.

In the simulation, the lower tube was fixed in all directions, while the upper tube was subjected to a shortening of 3.6 mm in the y direction. The rotational speed of the upper tube was set to 1000 rpm. According to Figure 6 for effective meshing of the post-deformation in the weld zone, each part was divided into two areas. The two areas close to the weld zone were meshed finely with a size of 0.08 mm, while the other areas were meshed coarsely with a size of 1.5 mm. The element size of 0.08 mm was determined through a mesh sensitivity analysis. The optimum mesh size was selected to be sufficiently fine so that the final temperature distribution was in agreement with the experimental results, and it also was no longer dependent on the further changes in the mesh size. Moreover, due to the use of a mapping solution technique, the mesh and aspect ratio were refined every 0.5 s during the simulation. The elements have a quadratic or triangular shape and the free technique was utilized for meshing. In this simulation, continuous reduced-integration elements (CGAX4RHT), which are well-suited for problems involving axial symmetry and coupled temperature–displacement, were used [11,15,23,24,44,45,46]. The element type used in the simulation has a degree of freedom for twist, which enables considering rotation and shear deformation out of the plane. This is crucial for the simulation of the RFW process. To handle the incompressible nature of the material during compression and plastic forming, a hybrid formulation was used [19,46].

## 3. Results and Discussion

### 3.1. Validation of the Thermal–Mechanical Simulation

**Figure 7:**
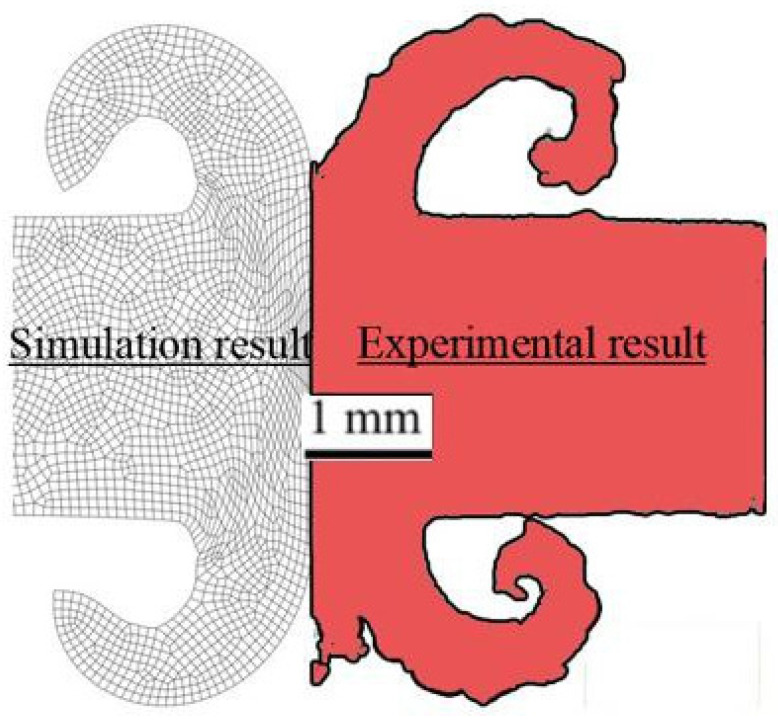
Comparison of the flash formation in the RFW process of the Inconel 718 alloy regenerated from experimental results [17] and obtained from simulation results.

In the simulation of the RFW process, the accurate definition of the initial conditions is imperative [16]. Consequently, the validation of this process is essential to determine whether the real-world system or process being simulated is accurately reflected by the simulation model. It is generally required to validate the variables of thermomechanical simulations with experimental test results. To ensure the accuracy of a simulation, one effective strategy is to juxtapose the deformed area observed in a practical process with its counterpart in the simulation [11,14,15,44,47]. This comparison allows researchers to scrutinize the similarities and discrepancies between the two. The insights gleaned from this analysis can then be used to evaluate the validity of the simulation. If necessary, adjustments can be made to enhance the accuracy of the simulation, thereby ensuring a more faithful representation of reality. In simulations of material deformation, the shape of the deformed region is influenced by several factors, including the precise definition of the material model and properties, the implementation of suitable boundary conditions, and the delineation of friction conditions. Ensuring that these parameters are correctly defined in the simulation is vital for obtaining accurate results. Cross-sections of the final shapes of the deformed flash area obtained from the practical test and predicted by the FE simulation are compared in Figure 7. This comparison provides valuable insights into the accuracy of the numerical simulation, and two key findings can be described based on the illustration in Figure 7. The experimental geometry of the flash formed towards the outside of the welded parts, as an important and fundamental criterion of an acceptable FRW process, has a good agreement with the geometry predicted by the FE simulation. Also, despite the severe plastic deformation of the flash region, the elements in the simulation results do not exhibit severe and abnormal distortion. This is because of using the remeshing technique, which helps to maintain the integrity of the mesh and prevent excessive distortion. As shown in Figure 7, a thickness of about 1 mm of the tubes is subjected to plastic deformation, which can be observed in both the experimental and simulation results.

**Figure 8:**
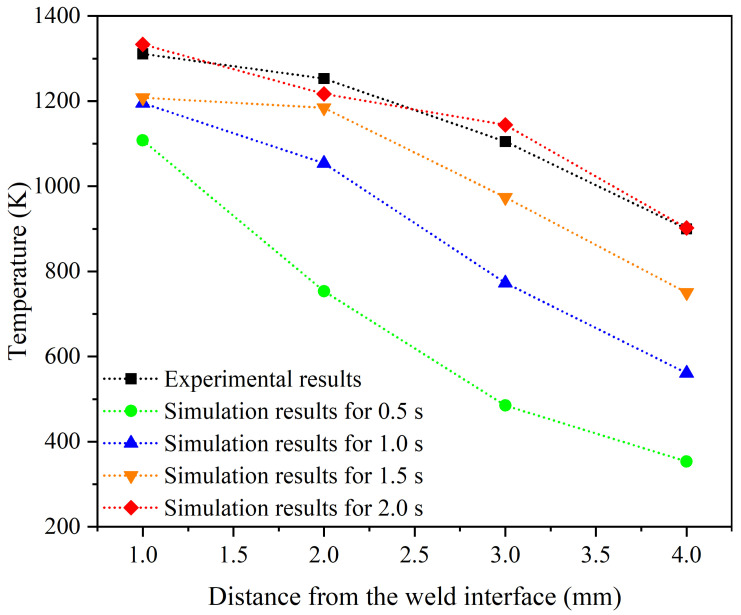
Comparison of temperature changes from practical test results [17] and simulation result during the RFW process of alloy 718 at different distances from the weld interface in 0.5, 1, 1.5, and 2 s.

To ensure the accuracy of temperature calculations in a thermomechanical simulation, researchers use thermocouples to measure temperature changes in experimental tests [15,16,24,44]. By comparing the experimentally measured results with the predictions of numerical simulations, the validity of the calculations can be assessed and any necessary adjustments to improve the accuracy of the simulation results can be conducted. The temperature measurement in the practical test of the RFW process of the Inconel 718 alloy tubes was performed using four thermocouples at different distances from the welding interface [12]. The measured results are compared with the simulation results of the current research in Figure 8. By analyzing the temperature changes as a function of distance from the welding interface, it is possible to predict the temperature distribution and determine different thermal zones of the welding process and grain size. Also, this information can be used to improve the welding process and ensure that the desired microstructure is achieved. The accuracy of the predictions can be assessed by comparing the red and black points in Figure 8. The other data points (temperatures in 0.5, 1, and 1.5 s) were extracted from the simulation just to show that the temperature changes as the time of the process passes. Also, due to possible errors in measuring the temperature, particularly in areas close to the weld interface, the results are predicted with reasonable accuracy, i.e., less than 50 °C which is acceptable when predicting temperatures exceeding 1000 °C. This comparison provides valuable insights into the validity of the thermal simulation and can help to identify any discrepancies that need to be addressed.

### 3.2. Thermal–Mechanical Simulation Results

Having significant deformation caused by softening due to an increase in temperature at the welding interface, it is important to study the thermomechanical evolutions in the RFW process through simulation. The results of numerical simulations, including key thermomechanical parameters such as stress and plastic strain components, temperature, and strain rate, can provide valuable insights into the behavior of materials during the RFW process. For example, by analyzing the changes in these parameters over the process time, engineers can gain a better understanding of how the material deforms and how its properties change during the welding process. This information can be used to optimize the welding process and improve performance and quality of the resulting welds.

**Figure 9:**
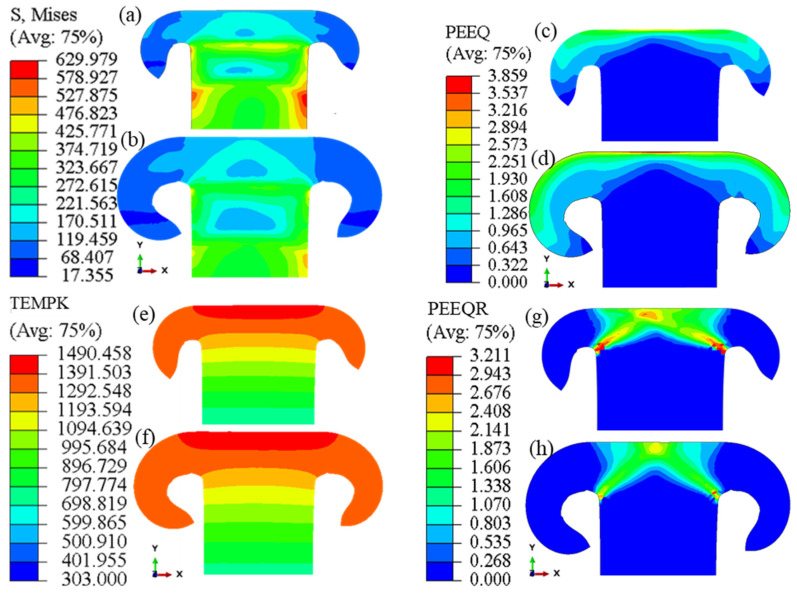
Distribution of effective stress, in MPa (**a**,**b**), equivalent plastic strain (**c**,**d**), temperature in Kelvin (**e**,**f**), and equivalent strain rate (**g**,**h**) at 1 and 2 (s) obtained by simulation of the RFW process.

As depicted in Figure 9, the region near the weld interface with the highest temperature exhibits the lowest magnitude of stress, except for the flash, which is not considered a part of the weldment. As the distance from the interface increased and the temperature decreased, the stress value increased accordingly. Figure 9b,h demonstrate that an increase in the strain rate of the material leads to an increase in the magnitude of flow stress. Figure 9 further illustrates that the stress in the center of the tube wall is higher than that in the welding flash. A significant decrease in stress near the weld interface, where temperatures are elevated, can result in the formation of the flash and flow of material from the weld interface toward free surfaces. This flow must be carefully considered when analyzing material behavior during the RFW process to ensure obtaining accurate simulations. The failure to achieve the desired increase in the temperature and stress reduction near the welding interface results in insufficient plastic deformation in this area, causing the tube to bend and fail at a far distance from the interface. Overall, the interaction between temperature, stress, and strain rate are prominent keys to insights into material behavior during the RFW process.

The quality of a weld is often assessed by the magnitude of plastic deformation at the weld interface. The formation of the flash and the presence of sufficient plastic deformation are deemed crucial indicators of the quality of a weld [48]. In the RFW process, a metallurgically clean weld joint can be created by the flow of material from the part’s interface, along with no oxides and surface contaminants [5]. As expected, the highest levels of strain were found to occur in areas with the highest temperatures, where softened material was extruded to form the flash. These conclusions demonstrate the importance of temperature and plastic deformation control in achieving high-quality welds in the RFW process.

In the RFW process, the accurate measurement of the temperature during experimental tests can be challenging. To overcome this, numerical simulation of the RFW process has been introduced as the most effective method for studying the temperature evolution [47,49]. It is known that 90% of heat generation in this process is attributed to the friction between two surfaces [20]. Therefore, an increase in the temperature can be anticipated as approaching the interface due to the friction. The temperature distribution resulting from the RFW process simulation is depicted in Figure 9e,f. According to the simulation, the maximum temperature at the weld interface was calculated to be 1217 °C. The computed temperature is lower than the melting point of the Inconel 718 alloy (that is, about 1400 °C), so the conception of solid-state conjoining in the RFW process has been observed in the simulation. The effectiveness of using simulations to study temperature evolution in the RFW process is demonstrated by these findings.

**Figure 10:**
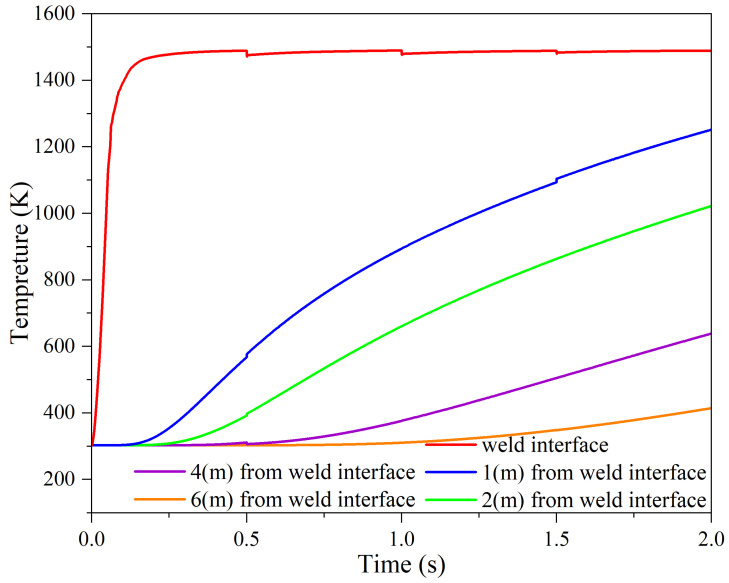
Temperature history curves for different sections with various distances from the weld interface at the end of the process obtained by simulation of the RFW process.

The study of temperature evolutions over time at different distances from the welding interface is crucial in identifying various zones within the welding heat-affected zone (HAZ) [50]. The measurement of these temperature changes is illustrated in Figure 10. With distance from the welding interface, an instant decrease in temperature is expected, and both the slope of the temperature increase and the maximum temperature are smaller. In this simulation, it was calculated that the appearance of plastic deformation in the welding interface occurred at approximately 0.12 s, at a temperature of 1000 °C. The prediction of the temperature distribution led to the conclusion that all plastic deformation occurred in zones where the temperature exceeded 900 °C.

**Figure 11:**
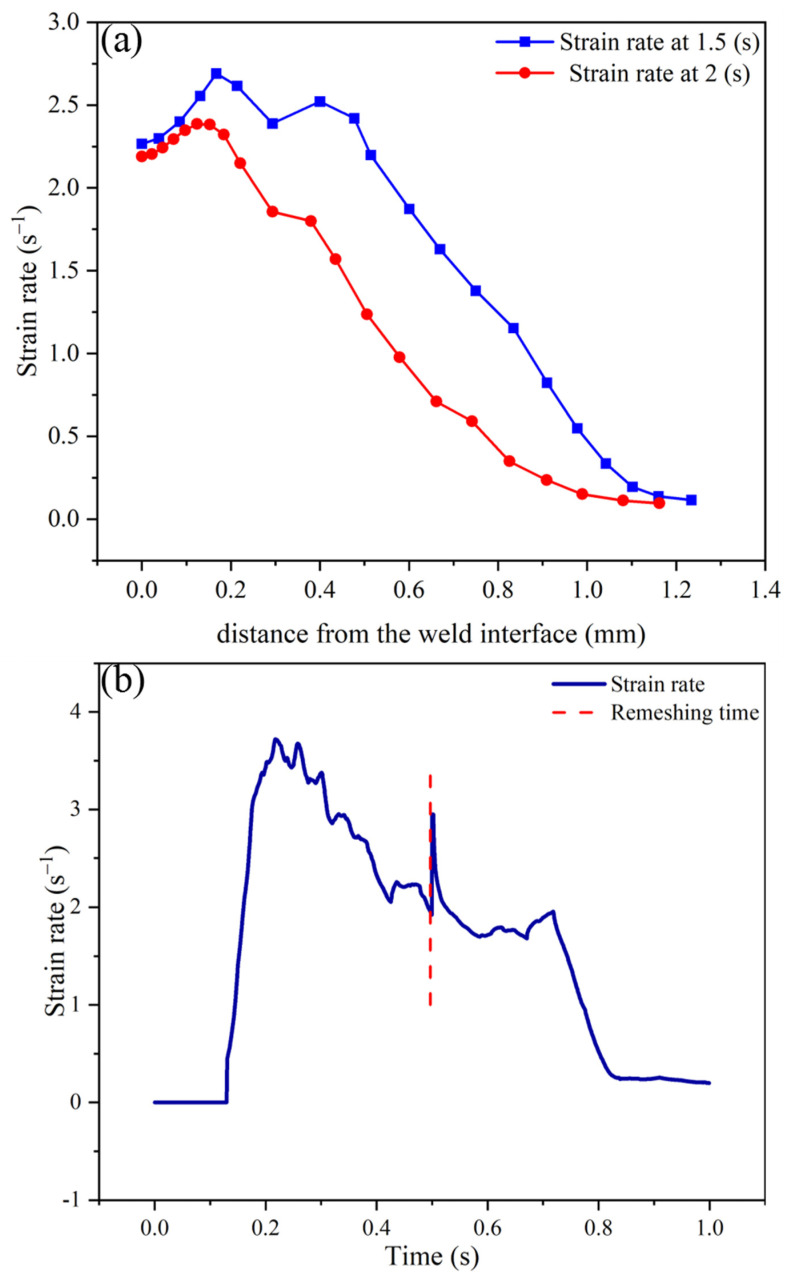
Strain rate distribution (**a**) with distance from the weld interface for different times and (**b**) across the weld interface vs. time.

In this section, the changes in the plastic strain rate are investigated by performing simulations for different parts of the welded tube. The simulations take into account the influence of the strain rate on the Zener–Hollomon parameter and dynamic recrystallization variables. According to Equations (5)–(11), calculating the evolution of the strain rate enables the computation of the Zener–Hollomon parameter, recrystallization volume fraction, and grain size, which can be used to simulate the microstructure evolution. Figure 11a presents the results of strain rate changes for different sections with various distances from the weld interface in the center of the tube wall thickness for 1.5 and 2 s. The reduction in the strain rate at the weld interface and distances away from it can be attributed to the adhesion of the material at the interface and the increase in strength of the material due to a decrease in temperature, respectively. To demonstrate this, Figure 11b shows the changes in equivalent strain rate across the weld interface at different times. According to this figure, as an element moves towards the flash, its strain rate decreases rapidly to become zero when entering the flash, which is also evident in Figure 9h.

By investigating the thermomechanical simulation results it was determined that the thermomechanically affected zone (TMAZ) was approximately 1 mm away from the weld interface. Additionally, the heat-affected zone (HAZ) was found to be approximately 12.5 mm away from the weld interface, which was determined by temperature distribution.

### 3.3. Validation of the Microstructural Simulation

Analyzing microstructural parameters yields crucial insights into the mechanical properties of parts fabricated using the RFW process, such as hardness and final strength [17]. This knowledge helps the design and manufacturing engineers, to enhance the performance and reliability of the final product using the RFW process. In this study, thermomechanical and microstructural simulations were conducted to deepen the understanding of these properties. During hot deformation processes, the free energy of an alloy increases due to the rise in density of dislocations, boundary area, and vacancies, leading to an unstable structure. These unstable structures tend to stabilize by reducing their energy through microstructural changes. Assessing this change can be used in determining initial RFW parameters to achieve suitable final properties. The driving force behind recovery and recrystallization phenomena is this energy reduction within the system. During the welding process of the Inconel 718 alloy, this reduction is primarily achieved through nucleation within the altered structure [36,51]. The JMAK kinetic equations are widely used to study microstructural evolutions related to nucleation and recrystallization during hot deformation processes [28,30,33,37]. These equations provide valuable insights into the mechanisms underlying these phenomena, allowing for improved understanding and control of the deformation process.

**Figure 12:**
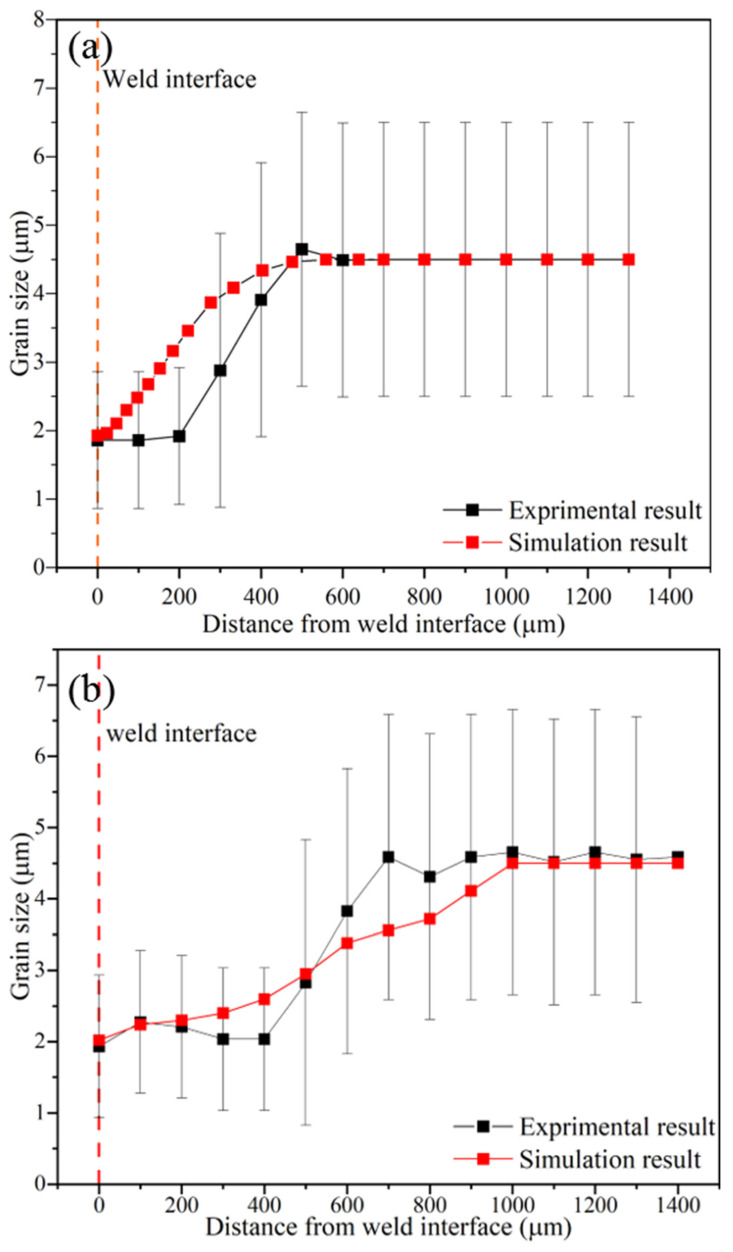
Comparison of grain size changes in micrometers obtained in the practical test [17] and simulation at the end of the RFW process (**a**) in the center and (**b**) at the edge of the tube wall.

In finite element simulation studies, validating results against practical test values is a crucial task for achieving a robust simulation algorithm. In this research, the microstructural simulation results were validated by comparing the calculated thickness of the recrystallized area with the values from experimental measurements. This comparison helps to ensure the accuracy and reliability of the simulation results, allowing for improved understanding and control of the microstructural evolution during the RFW processes. In the experimental tests [17], the thickness of the recrystallized area (as the distance from the weld interface) in the center and at the edge of the tube wall was reported to be 500 and 800 μm, respectively. In the simulation, the calculated thickness of the recrystallized area was 480 μm at the center of the tube wall and 800 to 900 μm at the edges. These results are consistent with experimental measurements, assuring the precision of the simulations. Figure 12 shows a comparison of grain size changes vs. the distance from the weld interface in the center and at the edge of the tube wall. The microstructural simulation results are shown to be acceptable by utilizing this method of comparison.

Deviations between simulations and experimental results can be attributed to errors in the simulation or potential inaccuracies in measuring the average grain size during experimental tests. To address the potential inaccuracies in measurements, the test results were assigned an error bar [17]. By conducting an accurate simulation, the majority of data points from the two curves depicted in Figure 12 fall within the range of the empirical test results. A simulation can predict the refinement of the structure near the welding interface due to an increase in the temperature. Elevated temperatures activate metallurgical mechanisms such as grain boundary migration, dislocation slip, and creep deformation, resulting in dynamic recrystallization and the formation of grains with smaller sizes than the initial grain sizes within the structure [36]. In summary, based on the validation, it can be concluded that by using thermal–mechanical simulation outputs as inputs for microstructural simulations, changes in grain size during the RFW process can be predicted.

### 3.4. Microstructural Simulation Results

**Figure 13:**
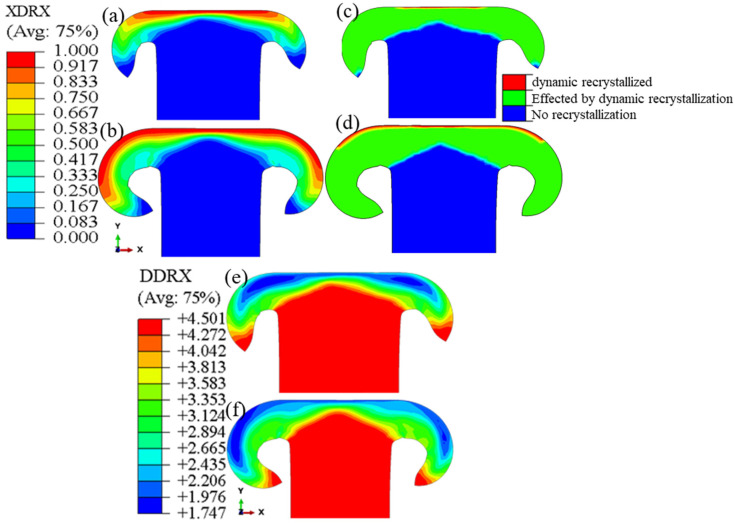
Distribution of volume fraction of recrystallization (**a**,**b**), distribution of different areas resulting from recrystallization (**c**,**d**), and average grain size distribution (**e**,**f**) at 1 and 2 (s) after the start of the process in the tube welded using RFW.

In this study, thermomechanical simulation was conducted to calculate the distribution of strain, strain rate, and temperature. The values were used in the JMAK equations to investigate the metallurgical parameters affected by hot deformation. To achieve this objective, microstructure simulations, by using Relations (7)–(13), were implemented into Abaqus via a user-defined subroutine. The results of the Johnson–Avrami recrystallization kinetic model simulation for 1 and 2 s are presented in Figure 13.

**Figure 14:**
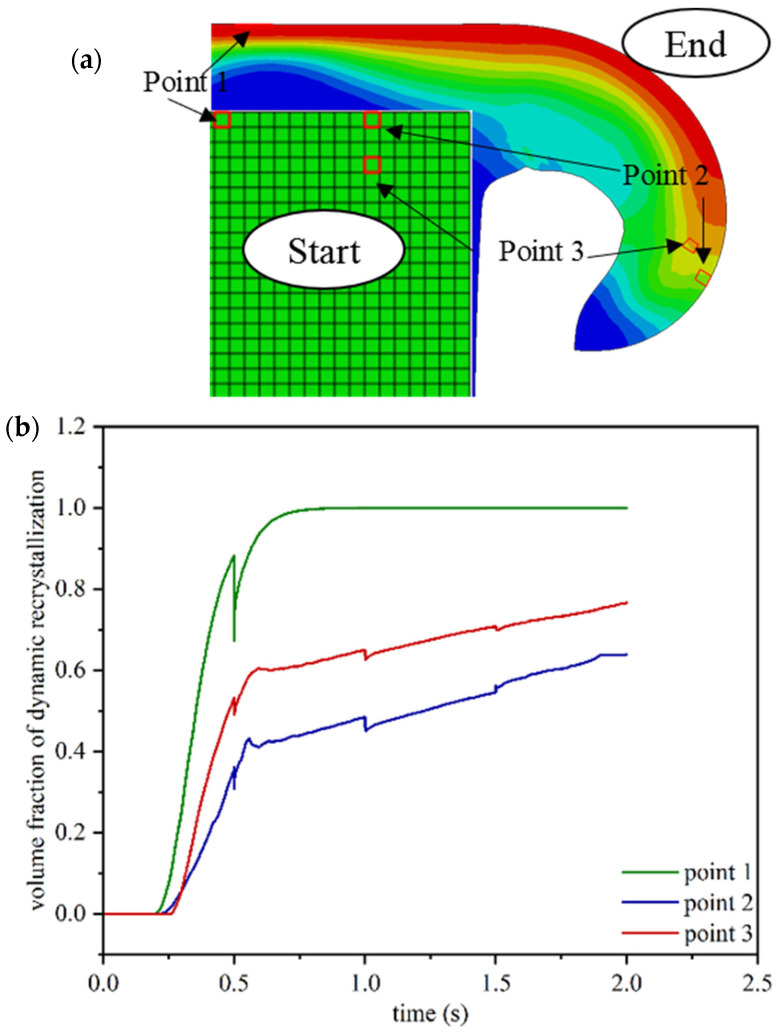
The position of the three points studied in microstructural simulation (**a**), and the recrystallization volume fraction changes in terms of time for the three specified points (**b**).

As shown in Figure 13, the highest amount of recrystallization volume fraction was calculated in the vicinity of the weld interface, where the temperature and plastic deformation are at their highest levels. Consistent with the impact of plastic strain stated by Equation (5), the calculated distribution of the recrystallization volume fraction was found to be similar to that of the plastic strain distribution. As depicted in Figure 13c,d, the fully recrystallized area (shown in red, with recrystallization exceeding 95%) within the depth of the weld interface expanded over time due to the increase in the temperature. By completion of the simulation, the thickness of the fully recrystallized area (indicated by the red region in Figure 13d) was determined to be approximately 0.1–0.2 μm for each part. In Figure 13c,d, which illustrate the distribution of different areas resulting from recrystallization, the initial formation of fully recrystallized areas (shown in red) within the center of the tube wall was calculated to occur at approximately 0.7 s. In regions that have experienced complete recrystallization, the microstructure is entirely dependent on the size of the recrystallized grains. To examine the evolution of recrystallization, the recrystallization volume fraction curves over time for three points are shown in Figure 14: one in the center of the tube wall on the weld interface (point 1), one close to the edge of the tube wall on the weld interface (point 2), and one close to the edge of the tube wall at a distance from the weld interface (point 3).

The thermomechanical simulation results indicate that the highest temperature and strain rate were predicted to occur in the center of the tube wall, as shown in Figure 9. Consequently, the simulation results based on microstructural equations revealed that the center of the tube wall had the highest volume fraction of recrystallization, as depicted in Figure 14, point 1. These observations are consistent with the expectations. During the entire process, point 1, located in the center of the tube wall, experienced the highest temperature and strain rate. As a result, it can be predicted that recrystallization will be completed in this area at the highest speed and in the shortest time.

The formation of a flash and the flow of materials into the weld flash resulted in a sharp decrease in the strain rate, as shown in Figure 11b. This decrease in the strain rate was accompanied by a corresponding reduction in the slope of the recrystallization volume fraction in points 2 and 3, as depicted in Figure 14. According to the curves shown in Figure 11a, the strain rate initially increases and then decreases with increasing distance from the weld interface. As a result, point 3 is located in an area with a higher strain compared to point 2. This higher strain rate at point 3 causes a greater volume fraction of recrystallization compared to point 2.

**Figure 15:**
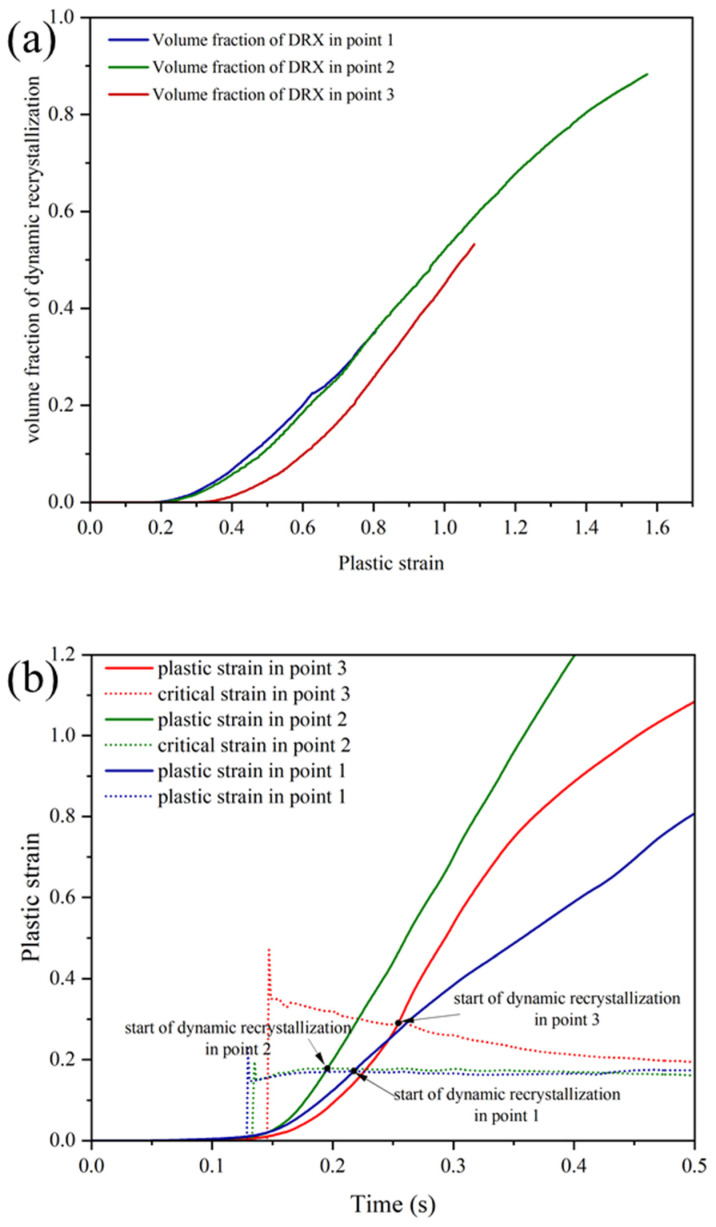
(**a**) Variations in recrystallization volume fraction vs. equivalent plastic strain and (**b**) variations in equivalent plastic strain vs. time for the three points of Figure 14.

According to relations 7 and 8, the critical strain and the strain for the 50% volume fraction of dynamic recrystallization (DRX) have an inverse relationship with the Zener–Hollomon parameter. As a result, it is expected that this parameter will be lower at points with a high temperature and a low strain rate. To illustrate the changes in critical strain over time, Figure 15b presents dashed curves for the points shown in Figure 14. For points 1 and 2, which are located at the welding interface and have the highest temperature, the lowest critical strain value was calculated.

**Figure 16:**
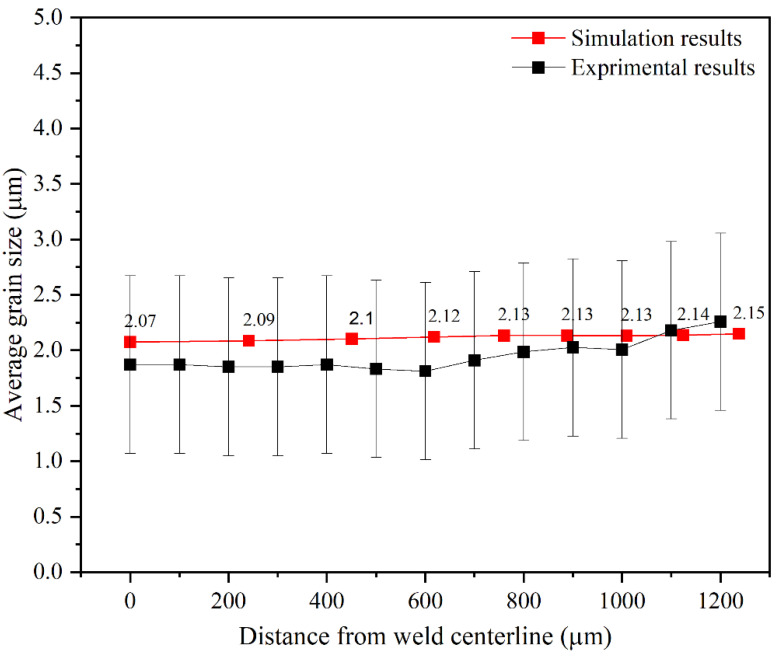
Grain size changes in micrometers at the weld interface obtained by the experiment and simulation.

At the end of the process, recrystallized grain size values were calculated for each element. The lowest average grain size value was found at points near the weld interface with the highest volume fraction of recrystallization, as shown in Figure 13. According to relation (10), there is a direct relationship between the grain size and the Zener–Hollomon parameter. Additionally, relation (9) states that an increasing temperature will cause the Zener–Hollomon parameter to decrease exponentially. As a result, there is an inverse relationship between temperature and the recrystallized grain size in each region, as shown in Figure 13e,f. Also, based on the microstructural simulation, the lowest value of the grain size was calculated to be in the vicinity of the weld interface, where the temperature is the highest. Similar to the experimental test results [17], the curve in Figure 16 indicates that a uniform grain size distribution along the welding interface can be predicted in simulations. As mentioned earlier, a uniform microstructure resulting from a uniform temperature distribution at the weld interface is considered one of the characteristics of high-quality weld formation in the RFW process.

## 4. Conclusions

In this study, sequential thermal–mechanical and microstructural simulation of the RFW process of the Inconel 718 alloy was performed. Calculations related to heat transfer, elastic and plastic deformation, and dynamic recrystallization of the microstructure during the welding process were resolved simultaneously. Simulation results including the distribution of plastic strain, temperature, strain rate, stress, recrystallization volume fraction, and final grain size of the Inconel 718’s structure were represented. The Johnson–Avrami relationships were used for microstructural simulation. The thermal–mechanical and microstructural simulation results were verified by comparison with experimental results to ensure the accuracy of the simulation. Furthermore, the effect of thermal–mechanical parameters on microstructural evolutions was assessed. Overall, the compatibility of simulation results with the experimental measurements shows that the formulations and physical mechanisms behind the obtained results can predict the thermal, mechanical and microstructural parameters in an acceptable accuracy. The summary of the findings is presented as follows.

1. The maximum value of effective plastic strain obtained from the thermal–mechanical simulation of the RFW process of Inconel 718 tubes with the assigned parameters was predicted as 3.86. Additionally, due to the friction between the welding faces, the temperature of the interface area increased to 1217 degrees Celsius. These results provide valuable insights into the changes in various parameters required for microstructural simulation.
2. The simulation accurately predicted high plastic deformation at the weld interface, which was caused by a decrease in material flow stress as a result of the increase in the temperature at the weld interface. Furthermore, the simulation provided valuable information on how microstructural parameters change, by predicting strain rate changes in different regions of the welded tube.
3. The distribution of the volume fraction of recrystallization was calculated using Johnson–Avrami relations, taking into account changes in plastic strain. Based on the results from solving the equations, the maximum amount of recrystallization volume fraction was found to occur in the center of the tube wall in the vicinity of the weld interface.
4. The thickness of the area affected by dynamic recrystallization phenomena was calculated by determining the recrystallization volume fraction at different points in the simulation. Accordingly, the thickness of this area was predicted as 480 μm at the center of the tube wall and 850 μm at the edge. These values were in close coherence with the values from experimental tests, which were reported as 500 and 800 μm, respectively.
5. In the same experimental test of the RFW process, the grain size near the welding interface was measured to be within a range of 1.9 to 2.2 μm. Correspondingly, the simulation predicted an average grain size of approximately 2 μm at the welding interface. These results demonstrate close agreement between the experimental measurements and the simulation predictions.

## Data Availability

Data are contained within the article.

## References

[1] Park G., Jeong S., Lee C. (2021). Fusion Weldabilities of Advanced High Manganese Steels: A Review. Met. Mater. Int..

[2] Pouranvari M. (2021). Critical review on fusion welding of magnesium alloys: Metallurgical challenges and opportunities. Sci. Technol. Weld. Join..

[3] Chamanfar A., Jahazi M., Cormier J. (2015). A review on inertia and linear friction welding of Ni-based superalloys. Metall. Mater. Trans. A.

[4] Ajay V., Babu N.K., Ashfaq M., Kumar T.M., Krishna K.V. (2021). A review on rotary and linear friction welding of Inconel alloys. Trans. Indian Inst. Met..

[5] Maalekian M. (2007). Friction welding—Critical assessment of literature. Sci. Technol. Weld. Join..

[6] Rangasamy S., Kamalamurthy S., Ponnusamy S., Bellamkonda P.N., Visvalingam B. (2023). Optimization of mechanical properties of rotary friction welding parameters of low alloy steel tubes using design of experiments concept. Int. J. Interact. Des. Manuf. (IJIDeM).

[7] Rehman A.U., Usmani Y., Al-Samhan A.M., Anwar S. (2021). Rotary friction welding of inconel 718 to inconel 600. Metals.

[8] Selvaraj R., Shanmugam K., Selvaraj P., Balasubramanian V. (2023). Optimization of process parameters of rotary friction welding of low alloy steel tubes using response surface methodology. Forces Mech..

[9] Nasution A.K., Gustami H., Suprastio S., Fadillah M., Octavia J., Saidin S. (2022). Potential use of Friction Welding for Fabricating Semi-Biodegradable Bone Screws. Int. J. Automot. Mech. Eng..

[10] Mousavi S., Kelishami A.R. (2008). Experimental and numerical analysis of the friction welding process for the 4340 steel and mild steel combinations. Weld. J..

[11] Li W., Shi S., Wang F., Zhang Z., Ma T., Li J. (2012). Numerical Simulation of Friction Welding Processes Based on ABAQUS Environment. J. Eng. Sci. Technol. Rev..

[12] Kuo C.-C., Gurumurthy N., Chen H.-W., Hunag S.-H. (2023). Experimentation and Numerical Modeling of Peak Temperature in the Weld Joint during Rotary Friction Welding of Dissimilar Plastic Rods. Polymers.

[13] Fu L., Duan L., Du S. (2003). Numerical simulation of inertia friction welding process by finite element method. Weld. J..

[14] Yang X., Li W., Fu Y., Ye Q., Xu Y., Dong X., Hu K., Zou Y. (2019). Finite element modelling for temperature, stresses and strains calculation in linear friction welding of TB9 titanium alloy. J. Mater. Res. Technol..

[15] Khosrowshahi J.H., Sadeghi M.H., Rasti A. (2020). Numerical simulation of plastic deformation in direct-drive friction welding of AISI 4140 and ASTM A106 steel tubes. Arch. Civ. Mech. Eng..

[16] Okeke S.I., Harrison N.M., Tong M. (2022). Computational modelling of dynamic recrystallisation of Ni-based superalloy during linear friction welding. Int. J. Adv. Manuf. Technol..

[17] Liu F.C., Nelson T.W. (2018). Grain structure evolution, grain boundary sliding and material flow resistance in friction welding of Alloy 718. Mater. Sci. Eng. A.

[18] Fanfoni M., Tomellini M. (1998). The Johnson-Mehl- Avrami-Kohnogorov model: A brief review. Il Nuovo C. D.

[19] Dassault-Systèmes (2020). Abaqus 2020 Analysis User’s Guide Volume II: Analysis. https://www.3ds.com/products-services/simulia/services-support/support/documentation/.

[20] Seli H., Awang M., Ismail A.I.M., Rachman E., Ahmad Z.A. (2013). Evaluation of properties and FEM model of the friction welded mild steel-Al6061-alumina. Mater. Res..

[21] Zhang Q., Zhang L., Liu W., Zhang X., Zhu W., Qu S. (2006). 3D rigid viscoplastic FE modelling of continuous drive friction welding process. Sci. Technol. Weld. Join..

[22] Uday M.B., Ahmad Fauzi M.N., Zuhailawati H., Ismail A.B. (2010). Advances in friction welding process: A review. Sci. Technol. Weld. Join..

[23] Wang F.F., Li W.Y., Li J.L., Vairis A. (2014). Process parameter analysis of inertia friction welding nickel-based superalloy. Int. J. Adv. Manuf. Technol..

[24] Jin F., Li J., Du Y., Nan X., Shi J., Xiong J., Zhang F. (2019). Numerical simulation based upon friction coefficient model on thermo-mechanical coupling in rotary friction welding corresponding with corona-bond evolution. J. Manuf. Process..

[25] Clas T., Ringius H. (2017). FE Modeling of Friction Welding Thermo-Mechanical Simulations Using ABAQUS. Master’s Degree.

[26] Singh S.K., Chattopadhyay K., Phanikumar G., Dutta P. (2014). Experimental and numerical studies on friction welding of thixocast A356 aluminum alloy. Acta Mater..

[27] Bai L., Wan S., Yi G., Shan Y., Pham S.T., Tieu A.K., Li Y., Wang R. (2021). Temperature-mediated tribological characteristics of 40CrNiMoA steel and Inconel 718 alloy during sliding against Si_3_N_4_ counterparts. Friction.

[28] Chen L., Sun W., Lin J., Zhao G., Wang G. (2019). Modelling of constitutive relationship, dynamic recrystallization and grain size of 40Cr steel during hot deformation process. Results Phys..

[29] Li C., Tan Y., Zhao F. (2019). Finite element simulation and process optimization of microstructure evolution in the formation of Inconel 718 alloy bolts. Mater. Res. Express.

[30] Razali M.K., Joun M.S. (2021). A new approach of predicting dynamic recrystallization using directly a flow stress model and its application to medium Mn steel. J. Mater. Res. Technol..

[31] Churyumov A.Y., Pozdniakov A.V. (2020). Simulation of Microstructure Evolution in Metal Materials under Hot Plastic Deformation and Heat Treatment. Phys. Met. Metallogr..

[32] Derazkola H.A., Garcia E., Murillo-Marrodán A., Fernandez A.C. (2022). Review on modeling and simulation of dynamic recrystallization of martensitic stainless steels during bulk hot deformation. J. Mater. Res. Technol..

[33] Quan G.-Z., Mao Y.-P., Li G.-S., Lv W.-Q., Wang Y., Zhou J. (2012). A characterization for the dynamic recrystallization kinetics of as-extruded 7075 aluminum alloy based on true stress–strain curves. Comput. Mater. Sci..

[34] Xu Y., Chen C., Zhang X., Dai H., Jia J., Bai Z. (2018). Dynamic recrystallization kinetics and microstructure evolution of an AZ91D magnesium alloy during hot compression. Mater. Charact..

[35] Quan G.-Z. (2013). Characterization for dynamic recrystallization kinetics based on stress-strain curves. Recent Developments in the Study of Recrystallization.

[36] Humphreys F.J., Hatherly M. (2012). Recrystallization and Related Annealing Phenomena.

[37] Lv Y.-P., Li S.-J., Zhang X.-Y., Li Z.-Y., Zhou K.-C. (2018). Modeling and Finite Element Analysis for the Dynamic Recrystallization Behavior of Ti-5Al-5Mo-5V-3Cr-1Zr Near β Titanium Alloy During Hot Deformation. High Temp. Mater. Process..

[38] Lenard J.G., Pietrzyk M., Cser L., Lenard J.G., Pietrzyk M., Cser L. (1999). Chapter 6—Microstructure Evolution and Mechanical Properties of the Final Product. Mathematical and Physical Simulation of the Properties of Hot Rolled Products.

[39] Sente Software JMatPro^®^. Retrieved 2020. https://www.sentesoftware.co.uk/jmatpro.

[40] Saunders N., Guo U.K.Z., Li X., Miodownik A.P., Schillé J.P. (2003). Using JMatPro to model materials properties and behavior. JOM.

[41] Saunders N., Guo Z., Li X., Miodownik A.P., Gu G. Modelling the Material Properties and Behaviour of Ni-Based Superalloys. Proceedings of the Superalloys 2004, the Minerals, Metals and Materials Society.

[42] Guo Z., Saunders N., Miodownik A., Schillé J.P. (2007). Quantification of High Temperature Strength of Nickel-Based Superalloys. Mater. Sci. Forum.

[43] Saunders N., Guo Z., Miodownik A., Schillé J.-P. (2008). Modelling high temperature mechanical properties and microstructure evolution in Ni-based superalloys. Sente. Softw. Intern. Rep..

[44] Geng P., Qin G., Zhou J. (2019). Numerical and experimental investigation on friction welding of austenite stainless steel and middle carbon steel. J. Manuf. Process..

[45] Bennett C., Hyde T., Williams E. (2007). Modelling and simulation of the inertia friction welding of shafts. Proc. Inst. Mech. Eng. Part L: J. Mater. Des. Appl..

[46] Lashgari H.R., Li S., Kong C., Asnavandi M., Zangeneh S. (2021). Rotary friction welding of additively manufactured 17-4PH stainless steel. J. Manuf. Process..

[47] Kessler M., Hartl R., Fuchs A., Zaeh M. (2020). Simulation of rotary friction welding using a viscoelastic Maxwell model. Sci. Technol. Weld. Join..

[48] Maalekian M., Cerjak H. (2009). Thermal-Phase Transformation Modelling and Neural Network Analysis of Friction Welding of Non-Circular Eutectoid Steel Components. Weld. World.

[49] Tang T., Shi Q., Lei B., Zhou J., Gao Y., Li Y., Zhang G., Chen G. (2022). Transition of interfacial friction regime and its influence on thermal responses in rotary friction welding of SUS304 stainless steel: A fully coupled transient thermomechanical analysis. J. Manuf. Process..

[50] Dzioba I., Pala T. (2020). Influence of LWE on Strength of Welded Joints of HSS S960—Experimental and Numerical Analysis. Materials.

[51] Damodaram R., Ganesh Sundara Raman S., Kalvala P. (2013). Microstructure and mechanical properties of friction welded alloy 718. Mater. Sci. Eng. A.

